# Combination of hydrophilic interaction liquid chromatography and top-down mass spectrometry for characterisation of adeno-associated virus capsid proteins

**DOI:** 10.1007/s00216-025-05874-4

**Published:** 2025-04-21

**Authors:** Corentin Beaumal, Felipe Guapo, Josh Smith, Sara Carillo, Jonathan Bones

**Affiliations:** 1 Characterisation and Comparability Laboratory, NIBRT – National Institute for Bioprocessing Research and Training, Foster Avenue, Belfield, Blackrock, Dublin, A94 X099 Ireland; 2 School of Chemical and Bioprocess Engineering, University College Dublin, Belfield, Dublin, D04 V1 W8 Ireland

**Keywords:** Adeno-associated virus (AAV), Top-down mass spectrometry (TD-MS), Electron transfer dissociation (ETD), Hydrophilic interaction liquid chromatography (HILIC), Ultraviolet photodissociation (UVPD), Gene therapy characterisation

## Abstract

Adeno-associated virus (AAV) viral vector-based gene therapy is advancing rapidly, offering potential treatments for rare and severe diseases. The AAV capsid consists of a combination of three viral proteins (VPs), VP1, VP2, and VP3, ranging from 59 to 81 kDa and present at a theoretical bulk ratio of 1:1:10. This study employed hydrophilic interaction liquid chromatography (HILIC) and mass spectrometry (MS) to achieve robust separation and detailed characterisation of AAV9 capsid proteins. Advanced top-down MS approaches combining multiple fragmentation techniques (HCD, ETD, EThcD, and UVPD) were successfully applied, increasing the sequence coverage up to 40% for VP3 and confirming N-terminal acetylation on VP1 and VP3. The workflow demonstrated high reproducibility between injection duplicates and was subsequently applied to the characterisation of in-house produced biological replicates of AAV9 samples from HEK293 cells, showing consistent results across them. Analysis of AAV9 derived from Sf9 insect cells, a more complex sample due to higher levels of modification of the capsid VPs, further evidenced method versatility. Overall, this study highlights the potential of HILIC-MS and advanced top-down MS approaches for detailed characterisation of AAV capsid proteins.

**Graphical Abstract:**

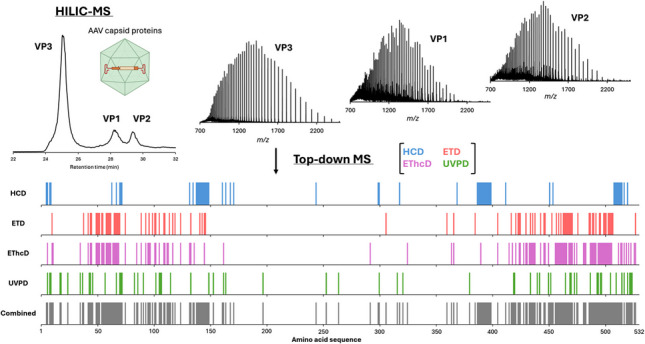

**Supplementary Information:**

The online version contains supplementary material available at 10.1007/s00216-025-05874-4.

## Introduction

Viral vector-based gene therapy is a rapidly advancing field with promising potential for treating rare and severe diseases [1–3]. This approach utilises selected vectors to deliver target genes to patients, either *in vivo* or *ex vivo,* with the goal of modifying or manipulating gene expression, or altering the biological properties of living cells for therapeutic use, often through the application of only a single dose to achieve long-lasting therapeutic effects [4, 5].

Among the commonly used vectors, adeno-associated virus (AAV) has gained considerable attention due to its favourable safety profile. As a non-pathogenic virus with primarily episomal expression and low integration frequency, AAV exhibits reduced immunogenicity compared to other viral vectors [6]. Wild-type AAV consists of a protein capsid encapsulating a therapeutic single-stranded DNA (ssDNA) genome (4.7 kb), flanked by two inverted terminal repeats (ITR) regions essential for genome replication and packaging. The open reading frames encoded within the capsid regulate replication (*rep*) and capsid protein synthesis and assembly (*cap*) [7–9]. Recombinant AAV (rAAV) vectors retain their ITRs, but *rep* and *cap* genes are replaced with the therapeutic gene of interest in either the ssDNA or in a double-stranded-self complementary (scDNA) conformation [7]. There are at least 12 wild-type serotypes of AAV identified in humans or primates, which differ in their capsid protein structure, thermal stability, and tissue tropism [10–12]. While the underlying mechanism for tissue tropism remains unknown, the AAV’s lifecycle has been explored considerably more [8, 13], implicating structural differences, the transgene, and capsid genetic content as potential causes for functional variability observed among serotypes and production systems [14–17].

The icosahedral capsid of AAV is built by a combination of approximately 60 copies of three viral proteins (VPs), VP1, VP2, and VP3, at an approximate bulk ratio of 1:1:10, respectively [6]. However, evidence of a divergent capsid assembly [18] and variability in VP incorporation across expression systems underscore the structural complexity of AAV capsids [14, 19, 20]. Such heterogeneity, along with their large capsid size, makes the characterisation of AAV-based products challenging. To address this, accurate analysis of bulk VP ratios, as well as the location and abundance of post-translational modifications (PTMs), is of utmost importance to aid understanding of the viral activity and to ensure high product safety and efficacy during rAAV manufacturing [21, 22].

**Fig. 1:**
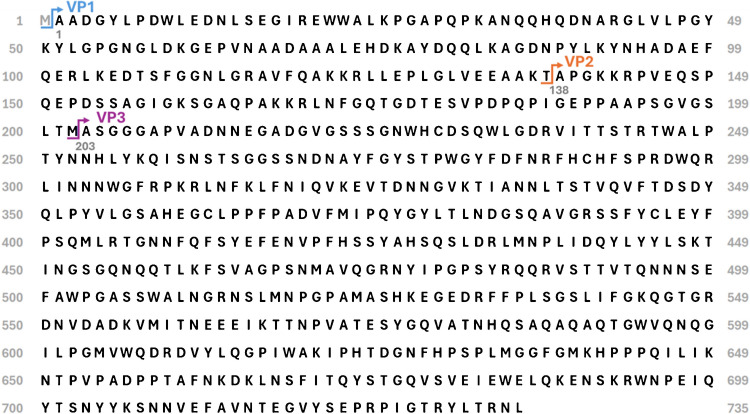
AAV9 capsid protein sequences. The blue arrow signifies the start of VP1, the orange arrow the start of VP2, and the purple arrow the start of VP3. The grey numbers convey the position of the amino acid residue above them in the VP1 protein sequence. Here, the amino acid residue considered to be the start of VP1 is the alanine residue with the grey 1 under it as the N-terminal methionine (grey M) is cleaved off in the cell

Liquid chromatography (LC) coupled to mass spectrometry (MS) has emerged as a powerful tool for the analytical characterisation of biotherapeutics [21, 23–25], and its high sensitivity makes it well suited for in-depth characterisation of samples of limited amount, like AAV-based gene therapy samples [23, 26, 27]. Most of the protein sequences, including the protein C-terminal, are shared between each VP (Fig. 1). Although bottom-up peptide mapping is an efficient way to fully characterise AAV capsid protein sequences, serotype identity, and identify PTMs [20, 28, 29], it fails to localise modifications located in each specific capsid protein when their location falls within their shared C terminal sequence. Alternative splicing gives VP2 an extra N-terminal domain compared to VP3, while VP1 possesses a further N-terminal domain, making it the largest capsid protein with a mass ranging from 80,336 to 82,106 Da depending on serotype [20, 30, 31].

Intact analysis of capsid protein by LC-MS, either under denaturing conditions [19] or followed by a denaturing protocol [20, 32], effectively addresses the challenge of separating VP1, VP2, and VP3. In most cases, this method enables comprehensive characterisation and determination of VP bulk ratios and PTM abundance [14, 19, 20, 30, 32]. When coupled with fluorescence-based detection, this enhances sensitivity further, making it suitable for analysing low-titre samples [27]. Following individual intact VP separation in denaturing conditions, top-down (TD) MS can be employed to fragment each intact proteoform, providing an alternative solution to the challenges encountered with peptide mapping. TD-MS approaches have already demonstrated their advantage over peptide mapping for characterisation of other biotherapeutics, such as determining the location of the payload in antibody-drug conjugates [33–36]. In addition, numerous studies point out the advantages of using multiple fragmentation techniques to increase sequence coverage and get complementary information [37–39]. While higher-energy collisional dissociation (HCD) was traditionally used, various fragmentation techniques, including electron transfer dissociation (ETD), electron transfer/higher-energy collisional dissociation (EThcD), or ultraviolet photodissociation (UVPD), are now fully implemented on commercially available instruments, facilitating combination of the different fragmentation techniques to obtain complementary specific information such as sequence variants or PTM location.

However, the large mass of VPs (from 59 to 81 kDa) complicates dissociation and full elucidation of their primary amino acid sequence when precursor ion fragmentation is achieved by HCD and ETD techniques [40]. By exploring current advancements in ion activation, sequence coverage analysis through top-down MS can be improved, facilitating deep interrogation of the primary structure of proteins to precisely localise mass discrepancies generally related to degradation events or PTMs within proteins [40]. Using multiple ion dissociation methods and manipulating product ions *via* gas-phase reactions, the feasibility of this application for AAV VP protein characterisation has been recently demonstrated, where the combination of multiple fragmentation methods along with proton transfer charge reduction achieved approximately 40% sequence coverage characterisation for the VP3 protein of AAV2 [41].

In the present study, we explore various fragmentation techniques available on the Thermo Scientific™ Orbitrap Eclipse™ Tribrid mass spectrometer to investigate top-down fragmentation of AAV9 VPs on the chromatographic timescale to determine sequence coverage and identification of PTMs. More specifically, we take advantage of highly informative fragmentation spectra to evaluate fragmentation repeatability across injection replicates and assess the benefits of combining multiple fragmentation techniques to build a solid foundation for using top-down for AAVs. Finally, the optimised method was applied to biological replicates from in-house produced AAV9 samples generated using the HEK293 cell line and then subsequently for the analysis of commercial AAV9 samples derived from the Sf9/baculovirus production system to explore potential differences between production systems.

## Materials and methods

### Chemicals and reagents

All reagents and solvents used were ACS reagent grade or better. Difluoroacetic acid 98% (DFA) was purchased from Arcos Organics (Fisher Scientific, Dublin, Ireland). Optima LC−MS grade acetonitrile (ACN), Thermo Scientific UHPLC-MS-grade water, Gibco™ Viral Production Medium (VPM), Gibco™ GlutaMAX™ Supplement (GlutaMAX™), Gibco™ Viral‑Plex™ Complexation Buffer, the Gibco™ AAV-MAX Transfection Kit, Gibco™ AAV-MAX Lysis Buffer (Lysis Buffer), and Gibco™ Pluronic™ F- 68 Non-ionic Surfactant (100X) (PF- 68) were all sourced from Fisher Scientific (Dublin, Ireland). The Gibco™ AAV-MAX Transfection Kit contains the Gibco™ AAV‑MAX Transfection Reagent, Gibco™ AAV‑MAX Transfection Booster, and Gibco™ AAV‑MAX Enhancer used for transfection. All other chemicals or solvents were obtained from Merck Sigma (Wicklow, Ireland).

### AAV9 production and purification

AAV9 samples from two different production systems were used. AAV9 from a HEK293 cell line production system was produced and purified in-house as described below. AAV9 (empty and full mix) from a Sf9 cell line production system was purchased from Virovek (Hayward, CA, USA) at a concentration of 2 × 10^13^ viral particles per mL (vp/mL).

#### AAV production and harvest

Gibco™ Viral Production Cells 2.0 (VPCs 2.0; Thermo Fisher Scientific Cat. No. A49784), a clonal derivative of the HEK293 F cell line, were maintained in suspension in VPM supplemented with 4 mM GlutaMAX™ for four passages. The incubation conditions for the cells were as follows: temperature, 37 °C; CO_2_, 8%; humidity, 80%; shake speed (25-mm orbital diameter), 120 rpm. On the final passage before transfection, cells were seeded in a 1 L shake flask at a viable cell density (vcd) of approximately 0.6 × 10^6^ cells/mL with a final culture volume of 240 mL. Three days after seeding, triple transfection was performed. The plasmids used for transfection were pAAV-GFP, pAAV2/9, and pAdDeltaF6. pAAV-GFP was a gift from John T. Gray (Addgene plasmid # 32395) while pAAV2/9 and pAdDeltaF6 were gifts from James M. Wilson (Addgene plasmid # 112867 and 112865, respectively) [42].

Triple transfection was performed following the procedure described in the AAV-MAX Helper-Free AAV Production System User Guide. Briefly, on the day of transfection, the VPCs 2.0 were diluted with fresh VPM supplemented with 4 mM GlutaMAX™ to a final vcd of 3 × 10^6^ cells/mL and used to generate three 120 mL cultures in 500 mL shake flasks, each with a vcd of 3 × 10^6^ cells/mL. 1.2 mL of AAV-MAX Enhancer (1:10 ratio Enhancer/culture volume) was added to each flask. The flasks were then incubated under the conditions described below, while the DNA/transfection complexation process was completed. DNA/transfection complexation was performed using 1.5 µg of total plasmid DNA/mL of culture. A plasmid molar ratio of 1:3:1 pAAV-GFP:pAAV2/9:pAdDeltaF6 was used. For each flask, the required amount of plasmid DNA was combined in a 15 mL tube and then diluted to a final volume of 12 mL (10% of the culture volume) with Gibco™ Viral‑Plex™ Complexation Buffer. The solution was then mixed by gently inverting the tube 3–5 times and then stored on ice. Next, 360 µL of AAV-MAX Transfection Booster (3 µL/mL of culture volume) was transferred to a clean 1.5 mL Eppendorf tube where 720 µL of AAV-MAX Transfection Reagent (6 µL/mL of culture volume) was added. The solutions were mixed by gently pipetting up and down 3–5 times. The AAV-MAX Transfection Booster/AAV-Max Transfection Reagent mixture was next added to the tube containing the diluted plasmid DNA. This complexion was then mixed by gentle inversion of the 15 mL falcon tube 3–5 times and incubated on ice for 20 min. After incubation, the transfection complexion was added to one of the incubating 120 mL cultures. This process was repeated for each of the 3 flasks transfected.

Seventy to seventy-two hours post-transfection, the AAVs from each flask were harvested. To harvest the produced AAVs, AAV-MAX Lysis Buffer was added to each flask at a 1:10 dilution. This was immediately followed by the addition of MgCl_2_ and Benzonase (Merck, Darmstadt, Germany) to final concentrations of 2 mM and 90 U/mL respectively. Cultures were then incubated for an additional 2 h under the previously described incubation conditions. After incubation, the contents of each flask were centrifuged at 16,000 × *g* for 20 min to remove the cell debris. The supernatants were then collected and stored at − 80 ℃ until ready for further downstream analysis.

#### AAV purification

Harvested material was affinity purified as described by Guapo et al. [14]. In short, samples were sequentially filtered through 0.45 µm and 0.2 µm PES membranes (Fisher Scientific, Dublin, Ireland) before purification to remove any remaining cell debris. Purification was performed using an ÄKTA Avant 150 FPLC system using 5 mL POROS™ GoPure™ AAVX pre-packed columns (0.8 × 10 cm) from Thermo Fisher Scientific (Paisley, UK). Loading was carried out on a flow rate of 1 mL/min, followed by two wash steps using 50 mM Tris, 1 M NaCl, 2 mM MgCl_2_, and 0.01% PF- 68 at pH 7.5 and 50 mM Tris, 0.2 M NaCl, 2 mM MgCl_2_, and 0.01% PF- 68 at pH 4.0 for 10 CVs each, respectively. Following equilibration with 50 mM Tris, 0.2 M NaCl, 2 mM MgCl_2_, and 0.01% PF- 68 at pH 7.5, elution of purified capsids was achieved with 80% 100 mM glycine, 2 mM MgCl_2_, and 0.01% PF- 68 at pH 2.0 for 10 CVs. The eluate was subsequently neutralised with 1.0 M Tris and then buffer-exchanged and concentrated in 0.01% PF- 68 in 1× PBS solution using 100 kDa Amicon^®^ Ultra- 0.5 filter devices (Merck, Darmstadt, Germany).

### Titre determination by SEC-LC-FLR

Size exclusion chromatography was carried out on a Thermo Scientific™ MAbPac™ SEC- 1 column (5µm, 2.1 × 150 mm, 300 Å pore size, Thermo Fisher Scientific, Sunnyvale, CA, USA), with a flow rate of 75 µL/min under isocratic conditions for 8 min with a buffer constituted of 50 mM sodium phosphate dibasic heptahydrate and 300 mM sodium chloride, pH 6.8. AAV titre determination was carried out based on triplicate measurements of peak areas quantified by fluorescence detection (excitation wavelength (*λ*_ex_) set to 280 nm, emission wavelength (*λ*_em_) set to 348 nm, and detector sensitivity set to 4) based on the assumption that capsids present elute at the exclusion limit for the SEC phase used. A standard curve was constructed by injecting different amounts (5 × 10^9^, 1 × 10^10^, 2.5 × 10^10^, 5 × 10^10^, 1 × 10^11^ vp/column, respectively) in triplicate of a reference sample of AAV of known concentration. Peak areas were integrated using a standard processing method on Thermo Scientific^TM^ Chromeleon^TM^ Chromatography Data System software version 7.2.10 ES. Using the calibration curve, the estimated capsid on a column of samples of unknown concentration was calculated following the fitting of a linear trendline with a correlation coefficient above 0.999. Capsid titre (vp/mL) could then be inferred using the quantitative experiment data and the volume injected.

### Instrumentation

Full-length AAV capsid protein separation was performed on a Thermo Scientific™ Vanquish™ Flex ultrahigh pressure liquid chromatography (UHPLC) instrument (Thermo Fisher Scientific, Germering, DE) consisting of a Quaternary Pump F (VF-P20-A), Split Sampler FT with 25 μL autosampler loop (VF-A10-A), Column Compartment H (VH-C10-A), and Fluorescence Detector F (VF-D50-A), coupled to an Orbitrap Eclipse™ Tribrid mass spectrometer through a standard flow Thermo Scientific™ Ion Max NG™ ion source containing a heated electrospray ionisation (H-ESI) probe (Thermo Fisher Scientific, San Jose, CA, USA). UHPLC instrument module settings were as follows: autosampler temperature set to 5 °C; column compartment temperature set to 25 °C; fluorescence detector excitation and emission wavelengths set to 280 nm and 348 nm, respectively, and detector sensitivity set to 4. Instrument modules were controlled with Thermo Scientific™ Xcalibur™ software version 4.7.

### Hydrophilic interaction chromatography (HILIC)MS and TD-MS experiments

VP separation of AAV9 was performed using an Acquity UPLC Glycoprotein BEH Amide Column, 300 Å, 1.7 μm, 2.1 × 150 mm (Waters Corporation, Milford, MA, USA) kept at 25 °C following a modified HILIC gradient [19]. Mobile phase A was UHPLC-MS grade water containing 0.1% (v/v) DFA, and mobile phase B was Optima LC−MS grade ACN containing 0.1% (v/v) DFA. Briefly, separation was performed at a flow rate of 0.1 mL/min, starting with an isocratic hold at 85% B for 3.5 min, followed by a decrease to 65% B over 0.1 min, and an isocratic hold at 65% B for 4.4 min. Then, a linear decrease from 65 to 58% B was performed over 21 min. The wash step was performed by simultaneously increasing the flow rate to 0.26 mL/min and decreasing mobile phase B to 0%, followed by a hold at 0% B for 3.5 min. Finally, column re-equilibration consisted of an increase to 85% B over 0.5 min, followed by an isocratic hold at 85% B and 0.26 mL/min for 5 min, and a decrease to 0.1 mL/min for 1 min. A volume of 1 µL and 1.5 µL of 1.5 × 10^14^ vp/mL (determined by SEC-FLR as mentioned in “Instrumentation” Section) AAV9 HEK293 (7 µL and 10 µL for AAV9 from Sf9) was injected for intact MS and top-down experiments, respectively. MS acquisition was performed using an Orbitrap Eclipse™ Tribrid mass spectrometer instrument equipped with UVPD and ETD/PTCR options. All the measurements were performed using intact protein mode at low pressure (1 mTorr in ion-routing multipole) and using the following source parameters: positive ion spray voltage was set to 3000 V, sheath and auxiliary gas were set to 25 and 8 (arbitrary units), respectively, RF lens was set at 125%, ion transfer tube temperature was 320 °C, and vaporiser temperature 150 °C. Full scan MS spectra were collected in profile mode at 15,000 resolution (at 200 *m/z*) using 5 microscans and a 700 to 2500 *m/z* mass range. AGC target set to 2 × 10^5^ and a maximum injection time to 100 ms. In-source CID dissociation energy was set to 35 V to assist with desolvation. Top-down MS^2^ experiments were performed in a targeted design by isolating a single charge state precursor ion in the quadrupole using a 5 *m/z* isolation window. All details of retention time windows, precursors charge states, and *m/z* are provided in Supplementary Table 1. Precursor ions were submitted to HCD, ETD, EThcD, and UVPD fragmentation. Resulting fragmentation spectra were recorded in profile mode without noise removal, using “full profile mode”. HCD used 20% and 30% normalised collision energies (NCE) based on the precursor charge state. ETD used 3 and 6 ms reaction time, and EThcD used the same reaction time with a supplemental activation energy of 15% NCE. The ETD reagent target was set at 7 × 10^5^ charges and the maximum ETD reagent injection time was 200 ms. UVPD used 15 and 30 ms activation time. All MS^2^ spectra were recorded at a resolution of 120,000 (at 200 *m/z*) using 5 microscans, with an AGC target set to 7.5 × 10^4^ and maximum injection time to 400 ms.

### Data processing and analysis

Full length AAV9 intact viral protein identification was performed in Thermo Scientific™ BioPharma Finder™ software version 5.2 (Thermo Fisher Scientific, San Jose, CA, USA). Briefly, raw data were deconvoluted using the ReSpect™ deconvolution algorithm with the sliding windows feature, applying a mass tolerance of 20 ppm for VP identification. Details about the deconvolution parameters can be found in Supplementary Table 2.

Processing of top-down data from various fragmentation methods was performed using BioPharma Finder (version 5.2) using the multiconsensus option. MS/MS spectra were deconvoluted using the Xtract™ algorithm included in BioPharma Finder software by applying a signal-to-noise ratio of 7, a fit factor of 80%, and considering only charge states 1+ to 25+. For fragment assignment, a mass tolerance of 10 ppm was applied. Fragmentation maps were obtained from ProSightBP (Proteinaceous, Evanston, IL, USA).

## Results and discussion

### AAV9 VP separation and intact characterisation

**Fig. 2:**
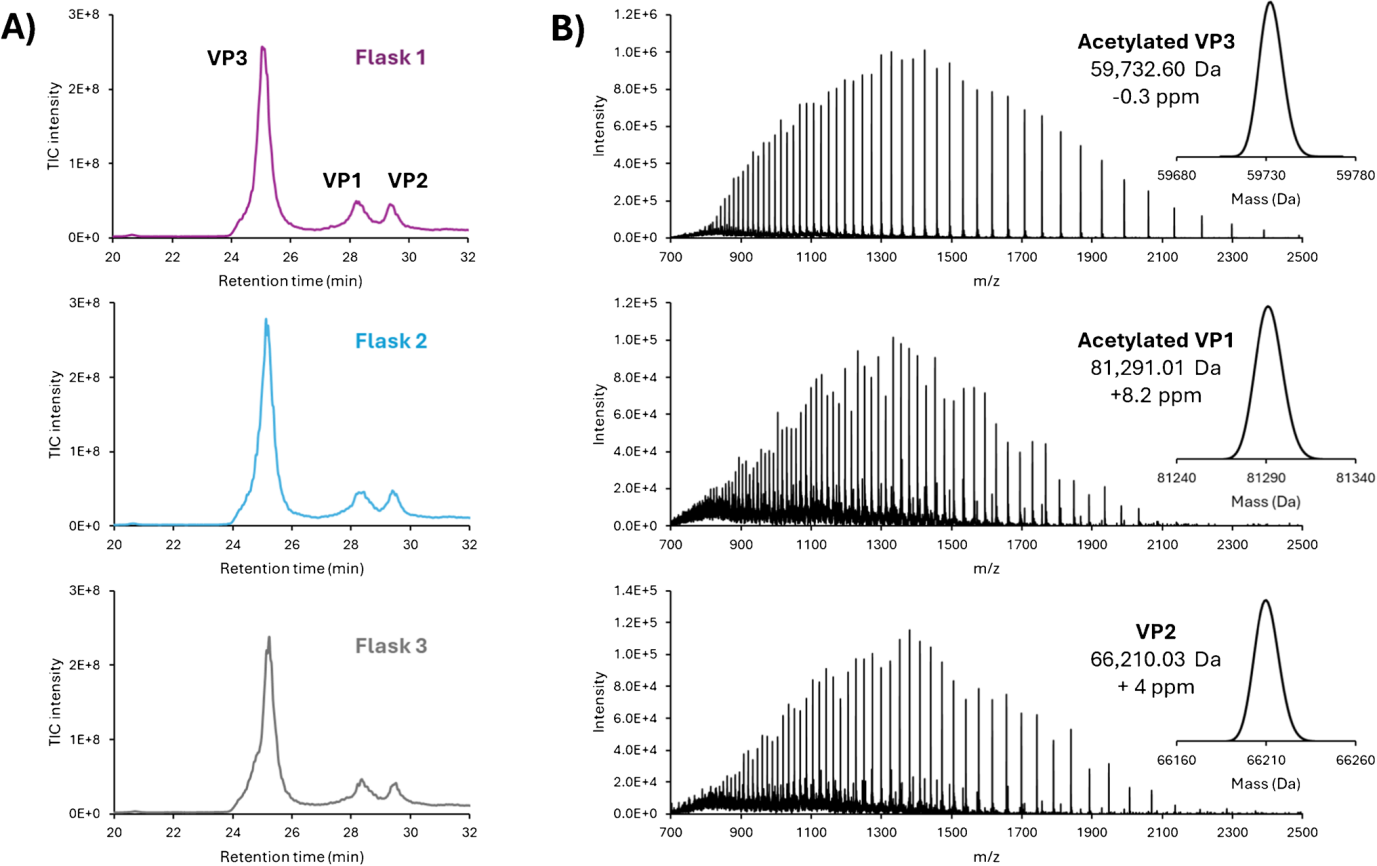
**A)** Total ion chromatogram (TIC) of AAV9 VP HILIC separation for the three biological replicates grown in parallel flasks 1–3. **B**) MS spectra of acetylated VP3 (top), acetylated VP1 (middle), and VP2 (bottom) from the biological replicate 1. Insets represent the deconvoluted spectrum, the experimental average mass, and the deviation from the theoretical average mass

Achieving effective separation of AAV VPs is required for determining the VP ratio and identifying potential PTMs. Multiple recent studies have demonstrated the advantages of using HILIC, with DFA as a mass spectrometry compatible mobile phase additive for comprehensive AAV VP separation [19, 20, 32]. In this study, we used a 30-min gradient to separate AAV9 VPs. As shown in Fig. 2A, the complete separation of VP3 was observed, while VP1 and VP2 were partially separated, with high-quality MS spectra obtained for each VP. VP ratio calculations were performed using two methods: FLR-based and MS-based. For the FLR-based approach, VP stoichiometry was calculated using the integrated area under the elution peak of each VP. For the MS-based approach, ratios between VPs were calculated using each VP’s area in the extracted ion chromatogram (XIC). In both cases, an approximate VP bulk ratio of 1:1:10 between VP1, VP2, and VP3 was found (Supplementary Table 3). This finding aligns with the expected theoretical ratio between capsid proteins for functioning AAV9 as documented in the literature [43]. Each VP exhibited a high-quality signal, which is crucial for accurate deconvolution. Notably, charge states 38+ to 95+ for VP1, 30+ to 77+ for VP2, and 24+ to 74+ for VP3 were observed and used for deconvolution. The results indicated excellent mass accuracy for the deconvoluted masses of each VP, with a mass error lower than 10 ppm for each (Fig. 2B). Furthermore, while VP2 was identified in its intact form, VP3 and VP1 were found to be acetylated (+ 42 Da). This observation is consistent with findings reported in other studies, whether for the AAV9 serotype, or for other serotypes [19, 30, 44].

### Top-down characterisation

**Fig. 3:**
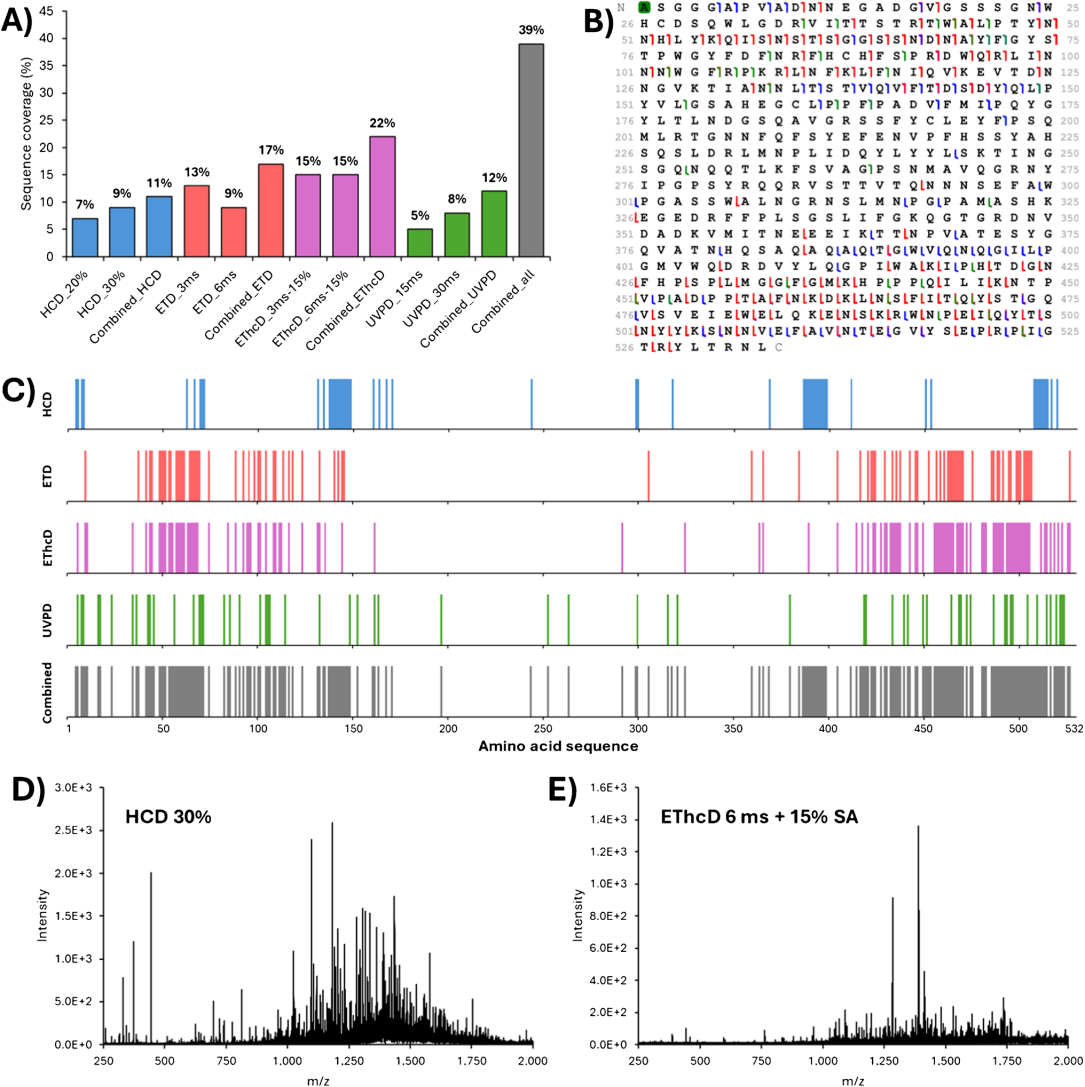
Sequence coverage of AAV9 VP3. **A**) Bar plot representing the sequence coverage obtained for each fragmentation, as well as the combination of the two runs from each technique (*n* = 2), and a combination of all the runs (*n* = 8). **B**) Combined fragmentation map of AAV9 VP3. **C**) Bar code representations of fragment location from HCD (blue), ETD (red), EThcD (pink), and UVPD (green) runs (*n* = 2 for each) and for the combination of all the runs (grey, *n* = 8) in the amino acid sequence. Fragmentation spectra of VP3 using **D**) HCD 30% NCE and **E**) EThcD 6 ms + 15% SA

Due to the high sequence homology among viral capsid proteins, their characterisation using a standard peptide mapping approach proves to be challenging [28, 45, 46]. To overcome this limitation, the combination of intact VP separation with TD-MS has emerged as a promising strategy. This approach allows for the characterisation of each individual VP and the identification of their potential PTMs [41]. Using the high reproducibility of chromatographic retention times, we developed a targeted method for each VP. A single charge state precursor was selected and then fragmented using various fragmentation techniques available on the Orbitrap Eclipse™ Tribrid instrument, including HCD, ETD, EThcD, and UVPD. We utilised two different activation energy/reaction times per fragmentation technique to induce fragmentation in different regions of the protein. As mentioned previously, the abundance and size of the VPs vary significantly, which can impact fragmentation efficiency. It is well established that the fragmentation of larger proteins is complex and often results in poor sequence coverage [47]. Here, VP3 (59.7 kDa) is smaller than VP2 (66.2 kDa) and VP1 (81.3 kDa) and has an intensity ten times greater than that of the other VPs, which enhanced fragment detection. The sequence coverage for each VP obtained from each run is illustrated in Fig. 3A and Supplementary Figure 1. Additionally, results from combining the same fragmentation technique (e.g., the two HCD runs) and of all the runs together (i.e., a total of 8 runs) are presented. The data indicates that fragmentation efficiency differs both between fragmentation techniques and among the different VPs. Specifically, VP3 exhibits the highest sequence coverage across all the techniques, achieving a sequence coverage of 39% when combining all the fragmentation techniques (*n* = 8 runs, Fig. 3B). In comparison, considering the same number of runs, sequence coverage for VP1 and VP2 is 5% and 10%, respectively. These observations align with the expected higher intensity of the VP3 precursor and its smaller size. Similarly, although the precursor abundances for VP1 and VP2 are comparable, the smaller size of VP2 facilitates fragmentation and leads to higher sequence coverage than observed for VP1.

Next, we examined the differences among the fragmentation techniques. For VP3, electron-based fragmentation (i.e., ETD and EThcD) yielded the best results, providing sequence coverage of 17% and 22% for ETD and EThcD, respectively, when combining results from the two runs for each technique. In addition, combining 2 HCD runs and 2 UVPD runs provided a sequence coverage of 11% and 12%, respectively. When considering all fragmentation techniques together for a single charge state (*n* = 8 runs), a total sequence coverage of 39% was achieved. These results are consistent with a recently published study on empty AAV2 samples using proton transfer charge reduction after fragmentation [41]. In contrast, for VP1 and VP2, HCD proved to be the most efficient fragmentation technique, yielding to sequence coverages of 3% and 5%, respectively (Supplementary Figure 1). The low sequence coverage observed for VP1 and VP2, particularly for electron-based fragmentations, can mainly be attributed to the low abundance of the precursor ion for these two proteins. This results in low-intensity fragment ions that are less likely to pass the S/N filter during data processing and annotation.

Another important aspect of this study is the benefit of using multiple fragmentation techniques and energies to improve the sequence coverage and gather information from the entire sequence of the VPs. Figure 3C represents the bar codes for each fragmentation technique and their combination for VP3. For HCD, only certain regions of the sequence are effectively fragmented (e.g., amino acids 135–150, 387–400, and 508–517), primarily due to the fragmentation pathway of this technique [48, 49]. Conversely, electron-based techniques (i.e., ETD and EThcD) exhibit a more random fragmentation pattern, with cleavage sites spread between amino acids 40 to 160 and 420 to 510. This difference between HCD and EThcD fragmentation is directly observed on the fragmentation spectra, as shown for HCD 30% NCE and EThcD 6 ms + 15% supplemental activation energy runs in Fig. 3D and E, respectively. Finally, UVPD generates fragment ions across the entire sequence without any fragmentation preference. In addition, the comparison of the fragment ions using a Venn diagram (Supplementary Figure 2) also highlights the need for multiple fragmentation techniques. Indeed, only 17 fragment ions (8% of all the identified sites) are shared by three or four techniques, while more than 50% of the fragment ions (111 fragment ions) have been only identified by a single fragmentation (36 by HCD, 14 by ETD, 33 by EThcD and 28 by UVPD). It can also be noted that 57 fragment ions are shared by ETD and EThcD, as expected regarding the similar pathway of these fragmentation techniques. The combination of each fragmentation techniques is therefore necessary to bring complementary information that enhances the overall sequence coverage of VPs, as evidenced by the combined bar code shown in Fig. 3C.

**Fig. 4:**
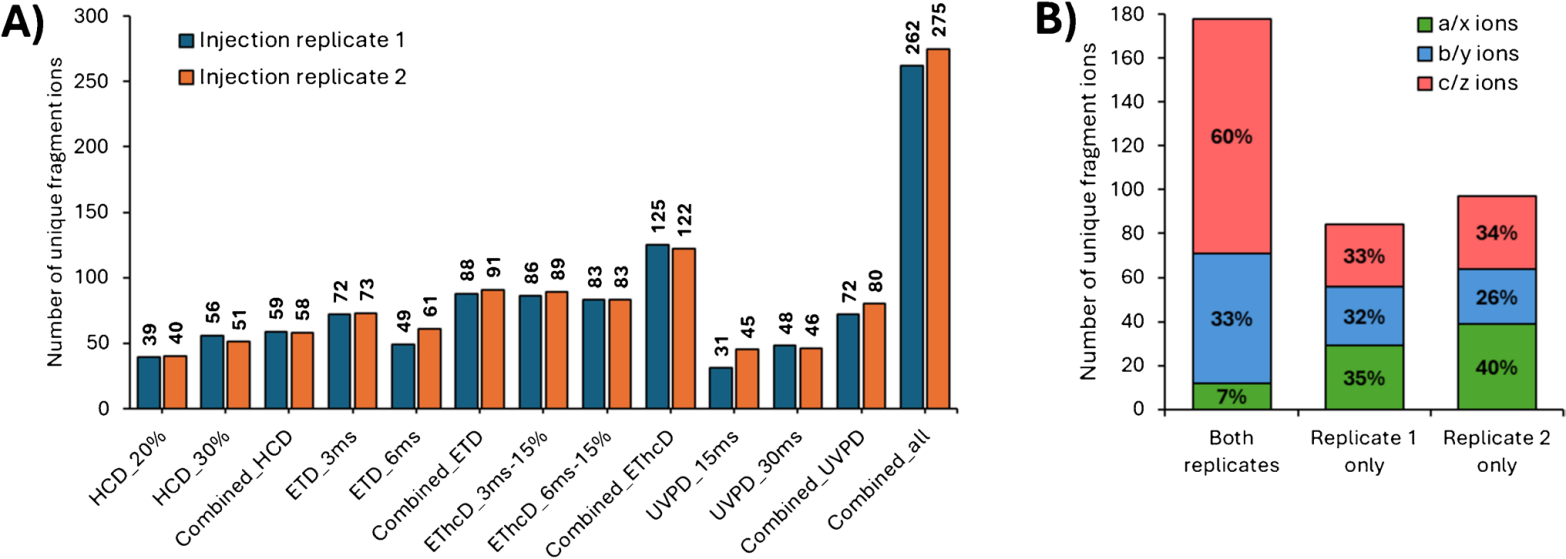
**A**) Number of unique fragments ions identified for each run, after combination of the runs from the same fragmentation technique (*n* = 2), and after combination of all the runs (*n* = 8) for injection replicates 1 and 2. **B**) Number of a/x (green), b/y (blue), and c/z (red) fragment ions found in both injection replicates, or only in one or the other injection replicates after combination of all the runs

To assess the repeatability of our approach, duplicate injections were performed for each fragmentation technique. The number of unique fragment ions and sequence coverage obtained from both injection replicates were compared, with results presented in Fig. 4. For most of the injection duplicates, the number of unique fragment ions identified was very similar, with less than 10% variation across duplicates. Only ETD 6 ms (20%) and UVPD 15 ms (31%) showed a higher variation. This higher variation in these two runs can be attributed to the smaller number of unique fragment ions identified (i.e., 49 and 61 for ETD 6 ms and 31 and 45 for UVPD 15 ms for replicates 1 and 2, respectively). When examining the combined results for each technique, it was found that the number of unique fragment ions remained close between the injection duplicates. Overall, high levels of repeatability were achieved using the method, with 262 unique fragment ions being identified in replicate 1 and 275 in replicate 2. Of these, 178 were shared between the two replicates, representing 68% and 65% of replicates 1 and 2, respectively (Fig. 4B). Among the ions found in both duplicates, 60% were *c* and *z* ions (mostly from ETD and EThcD), 33% were *b* and *y* ions (mainly from HCD), and only 7% were *a* and *x* (from UVPD), as depicted in Fig. 4B. Contrarily, unique fragment ions from UVPD (i.e., *a* and *x* ions) represent 35% and 40% of the unique fragment ions found only in injection replicates 1 and 2, respectively.

### Analysis of replicate in-house produced AAV9 using triple transfection in HEK293 cells

**Fig. 5:**
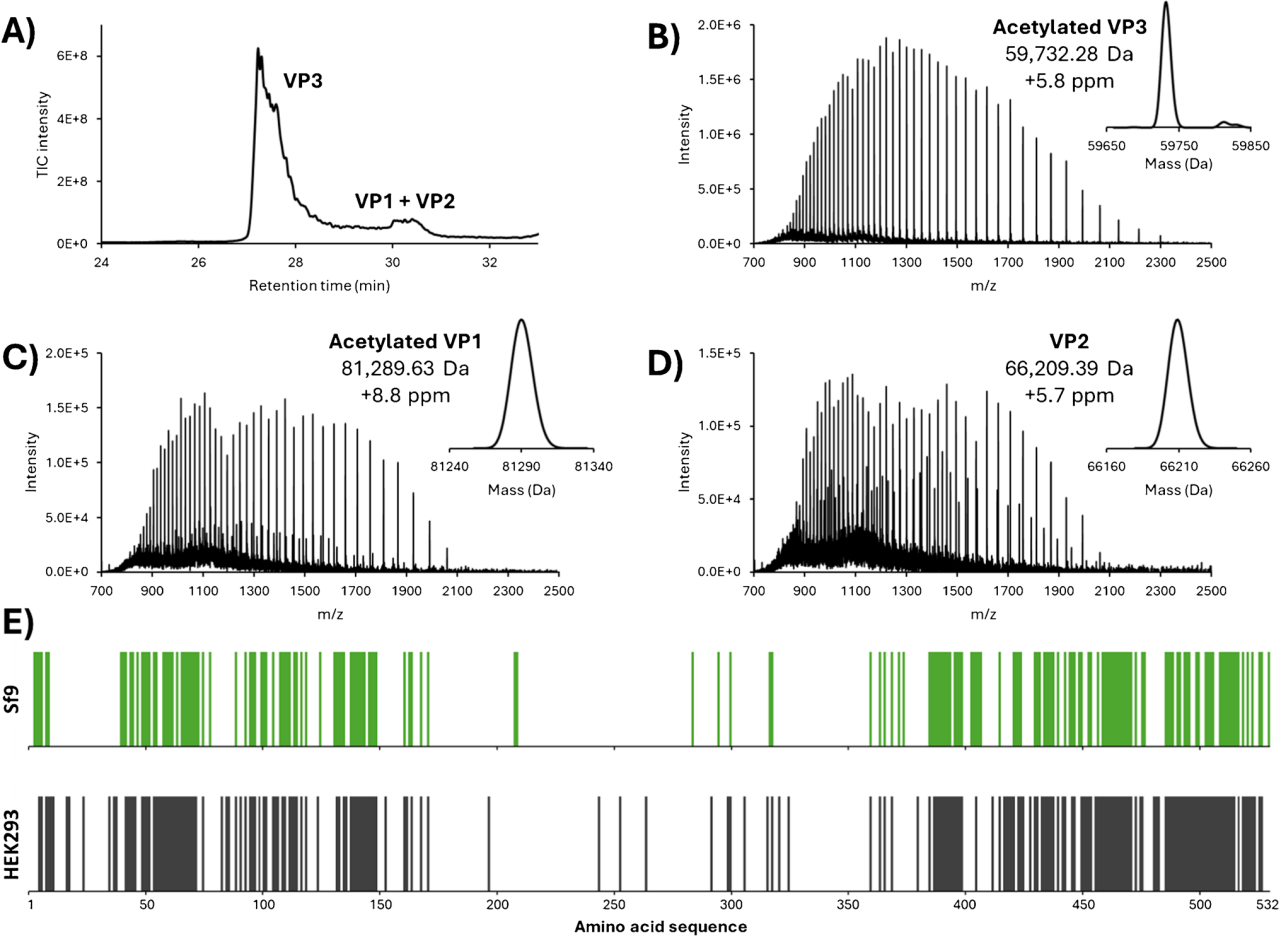
**A**) Total ion chromatogram (TIC) of AAV9 from Sf9 VP HILIC separation. MS spectra of **B**) acetylated VP3, **C**) acetylated VP1, and **D**) VP2. Insets represent the deconvoluted spectrum, the experimental average mass, and the deviation to the theoretical average mass. **E**) Bar code representations of fragments location for the combination of all the runs (grey, *n* = 8) for AAV9 samples from Sf9 and HEK293 production systems

Three biological replicates of AAV9 samples were independently produced in separate flasks and purified in-house. These three biological replicates were analysed using LC-MS and top-down approaches, as mentioned previously. The separation of the three VPs demonstrated a high reproducibility, with less than 1% retention time variation between the biological replicates (Fig. 2). The VP ratio for each of the biological replicates was compared using the FLR- and MS-based methodologies described above. For all replicates, the observed experimental ratio was approximately 1:1:10 for VP1:VP2:VP3, which aligns with expectations from previous literature and highlights the reproducibility of the AAV9 production method used (Supplementary Table 3). Subsequently, the three biological replicates were analysed in duplicates using the top-down approach described earlier. The results are presented in Table 1, Fig. 5, and Supplementary Figure 3. Specifically, for each flask, we observed an overall sequence coverage of 4–5% for VP1, 7–10% for VP2, and 36–40% for VP3. Moreover, sequence coverage between the injection duplicates of a single biological replicate and among the biological replicates themselves was comparable for all VPs (Table 1).

**Table 1:** Combined sequence coverage of AAV9 VP1, VP2, and VP3 for each biological and injection replicate

Combined sequence coverage	Biological replicate 1		Biological replicate 2		Biological replicate 3	
	Injection 1	Injection 2	Injection 1	Injection 2	Injection 1	Injection 2
VP1	5%	5%	4%	4%	4%	4%
VP2	10%	9%	7%	7%	7%	8%
VP3	39%	40%	36%	39%	38%	39%

Additionally, the detailed results for VP3 underline the consistency of our approach across different samples. The coefficient of variation is below 15% for all the individual fragmentations and the combined results regarding sequence coverage and number of unique fragments ions identified (Supplementary Figure 4. A and 4.B, respectively) across all replicates (i.e., including both biological and injection replicates). This variation is particularly low for HCD-based techniques, as fragmentation pathways appeared less random.

### Application to AAV derived from Sf9 insect cells

AAV production methods typically involve either the transient transfection of HEK293 cells or the recombinant baculovirus infection of the *Spodoptera frugiperda* (Sf9) cells. The HEK293 cell line is the most widely used system for AAV production but remains a high-cost approach and suffers from limited scalability [9, 50], while Sf9 production systems often lead to high yields [50, 51] and achieve high full/empty ratios at lower cost. Nevertheless, for Sf9, optimisations are required to achieve the desired 1:1:10 ratio of VP1:VP2:VP3 [9], and it has been noted that VPs generally have a greater number of PTMs and exhibit more truncated VP species [52, 53]. To explore the differences between the two expression systems, the method developed for the HEK293 cell line was applied to the AAV9 from the Sf9 production system. AAV9 empty reference material produced using baculovirus infections of Sf9 cells was purchased from Virovek (Hayward, CA, USA). We first performed an intact mass analysis of the AAV9 VPs. The results show a good separation of VP3 from other VPs, while VP1 and VP2 are poorly separated, making ratio calculation difficult (Fig. 5). For Sf9 samples, both VP1 and VP3 were found to be acetylated, while VP2 was found in its intact form, as observed for samples from HEK293. The mass error for all the VPs, when comparing the deconvoluted mass to the theoretical mass, was less than 10 ppm (Fig. 5). Several low-abundant species were also identified, corresponding to VP-bearing PTMs such as deamidation, phosphorylation, or oxidation. Although these species are present at low abundance, their existence may account for the low separation efficiency of the VPs and the broad peaks observed in the analysis. Fragmentation of the VPs using the same approach used for samples from HEK293 was also performed. For VP3, the sequence coverage from different fragmentation methods were as follows: 12% for HCD, 22% for ETD, 11% for EThcD, and 2% for UVPD, resulting in a combined sequence coverage of 33%, which is close to the 39% observed previously for HEK293 samples. Comparison of the bar code representations of both production systems confirmed the similarity of the fragmentation pattern, thereby strengthening confidence in our methodology (Supplementary Table 4 and Fig. 5E). The slightly lower combined sequence coverage observed for Sf9 compared to HEK293 might be explained by the possible co-isolation of other low abundant VP3 species precursors (e.g., phosphorylated VP3). This co-isolation could create signal interference, ultimately decreasing the overall signal-to-noise ratio. However, pinpointing phosphorylation on the sequence is extremely challenging due to the very low intensity of the phosphorylated VP3 fragment ions compared to the predominant VP3 species. To do so, a better separation of the different proteoforms would be required. In conclusion, this study on AAV samples from Sf9 demonstrates that our methodology is effective for analysing AAV samples from different production systems. Unfortunately, this approach remains limited for VP1 and VP2, with combined sequence coverage of 2% and 3%, respectively, likely due to their coelution and low abundance.

## Conclusions

This study successfully demonstrates a comprehensive approach to analysing of AAV9 VPs using HILIC-MS-based methods. The methodology enabled the effective separation of VP1, VP2, and VP3, confirming the theoretical 1:1:10 ratio for the capsid proteins. The combination of multiple fragmentation techniques (HCD, ETD, EThcD, and UVPD) on a single charge state proved to be beneficial and necessary for enhancing sequence coverage on the LC time scale and enabled characterising N-terminal acetylation on VP1 and VP3. Moreover, injection duplicates further validated the repeatability of the methodology, demonstrating minimal variability. It confirms the versatility of the approach and serves as solid foundation for future work on AAVs using top-down approaches. This work also highlights the consistency and the reproducibility of our TD-MS approach across biological replicates, as well as for AAV9 samples from different production systems. The study establishes a robust, versatile, and highly repeatable methodology that offers detailed insights into capsid protein characterisation. Our findings align with recently published literature on AAV2 [41], and additionally highlight the reproducibility achieved when considering biological replicates and production systems, making our approach available to serve as a solid foundation for in-depth VP analysis across other AAV serotypes. Notwithstanding, further optimisation of the LC method and implementation of additional ion-ion reaction techniques (e.g., proton transfer reaction) may be necessary to improve the sequence coverage and PTM location, particularly for low-abundance critical PTMs such as deamidation [54]. Currently, VP1 and VP2 detailed characterisation remains challenging, and single proteoforms characterisation remain for now a limitation due to their low abundance.

## Supplementary Information

Below is the link to the electronic supplementary material.

Supplementary file1 (DOCX 357 KB)

## Data Availability

The data generated and analysed in this study are available from the authors by request.
