# Supplementary material for: Combination of hydrophilic interaction liquid chromatography and top-down mass spectrometry for characterisation of adeno-associated virus capsid proteins
Source: Anal Bioanal Chem. 2025 Apr 21;417(15):3405–17. doi: 10.1007/s00216-025-05874-4 (PMC12122587; doi:10.1007/s00216-025-05874-4)
Supplement: Supplementary file 1 — Supplementary file1 (DOCX 357 KB) [file 216_2025_5874_MOESM1_ESM.docx]

**Combination of hydrophilic interaction liquid chromatography and top-down mass spectrometry for characterisation of adeno-associated virus capsid proteins**

Corentin Beaumal^a^, Felipe Guapo^a^, Josh Smith^a^, Nora Crushell^a^, Sara Carillo^a^, Jonathan Bones^a, b^.

^a^ Characterization and Comparability Laboratory, NIBRT – National Institute for Bioprocessing Research and Training, Foster Avenue, Belfield, Blackrock, Dublin, A94X099, Ireland

^b^ School of Chemical and Bioprocess Engineering, University College Dublin, Belfield, Dublin D04 V1W8, Ireland

Corresponding author: Jonathan Bones, [jonatahan.bones@nibrt.ie](mailto:jonatahan.bones@nibrt.ie).

**Supplementary information**

**
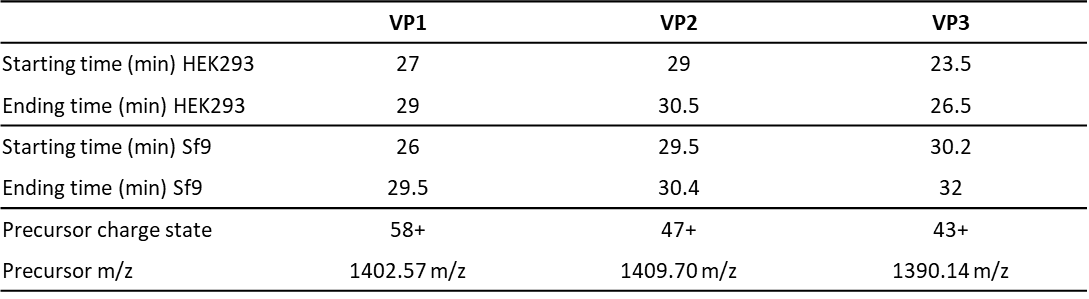
**

**Supplementary Table 1:** Acquisition times and precursors charge states and m/z of VP1, VP2 and VP3 used for fragmentation.

**
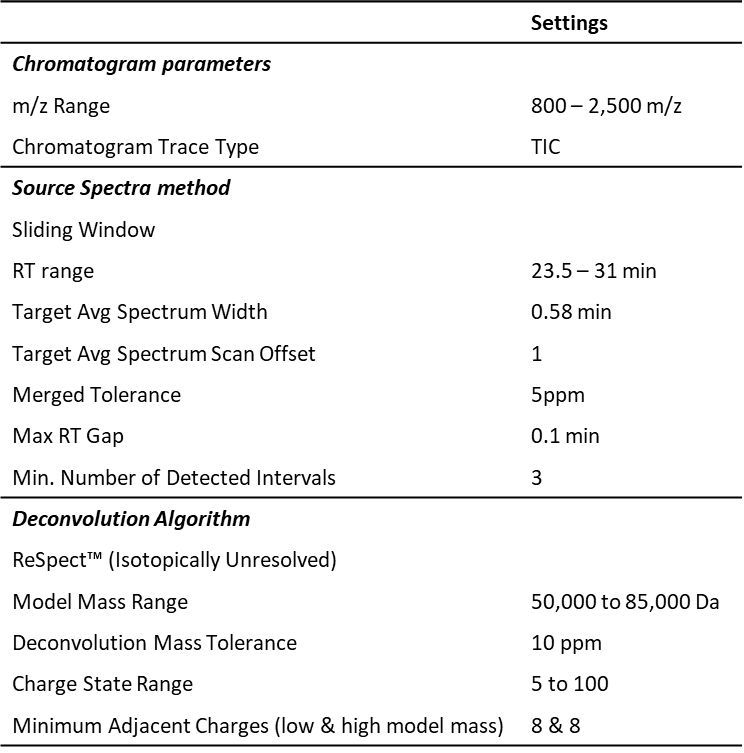
**

**Supplementary Table 2:** BioPharma Finder 5.2 deconvolution parameters for VPs intact mass determination.

**
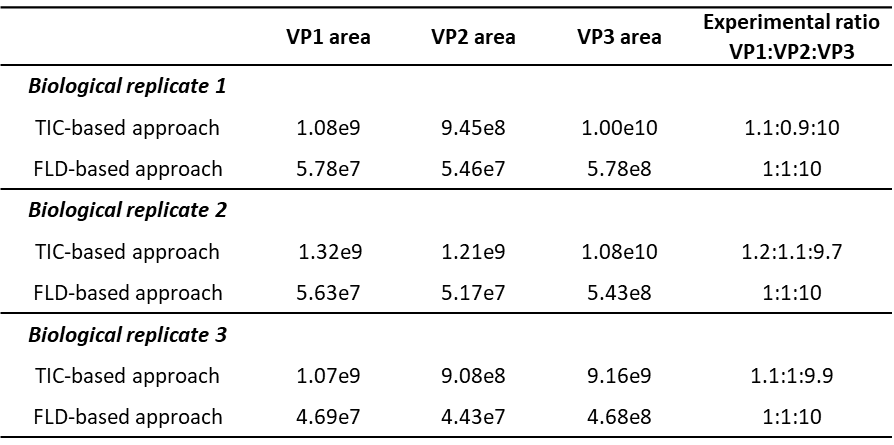
**

**Supplementary Table 3:** TIC and FLD areas from FreeStyle of each biological replicate, and the experimental ratio calculated from these values.

**
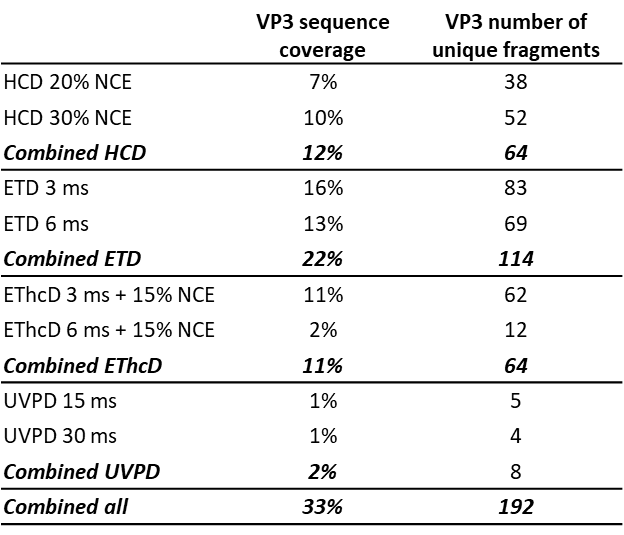
**

**Supplementary Table 4:** Sequence coverage and number of unique fragments ions identified for the VP3 of AAV9 from Sf9.

**
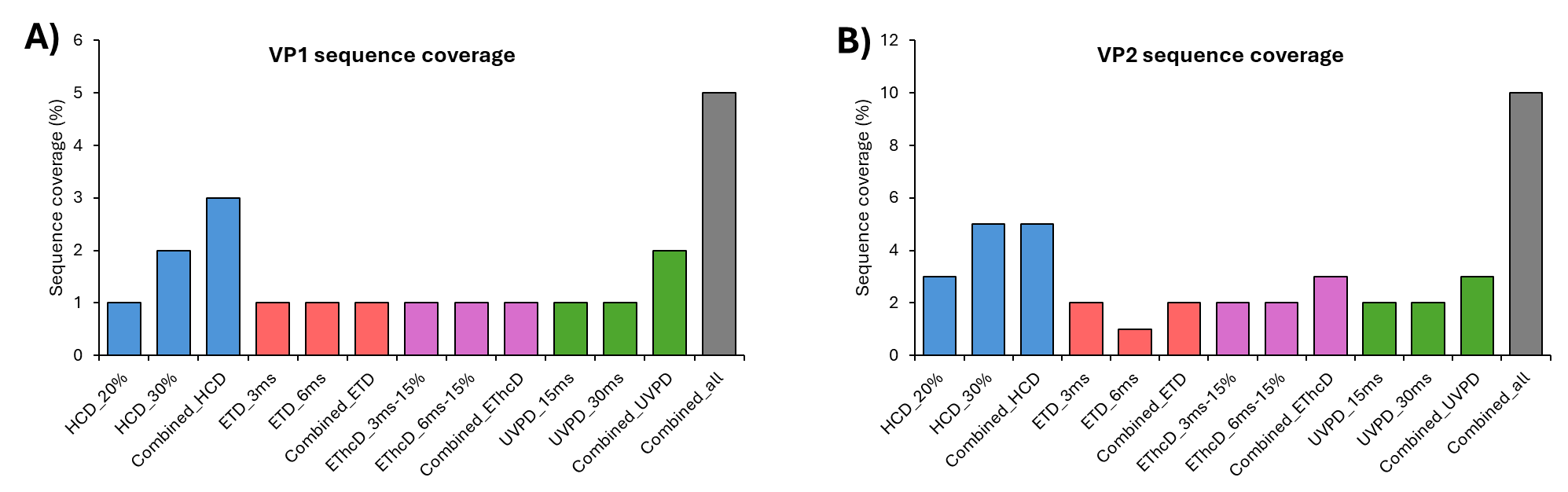
**

**Supplementary Figure 1:** Sequence coverage of (A) VP1 and (B) VP2 for each run, for each fragmentation technique (n = 2) and for the combination of all the runs (n = 8).


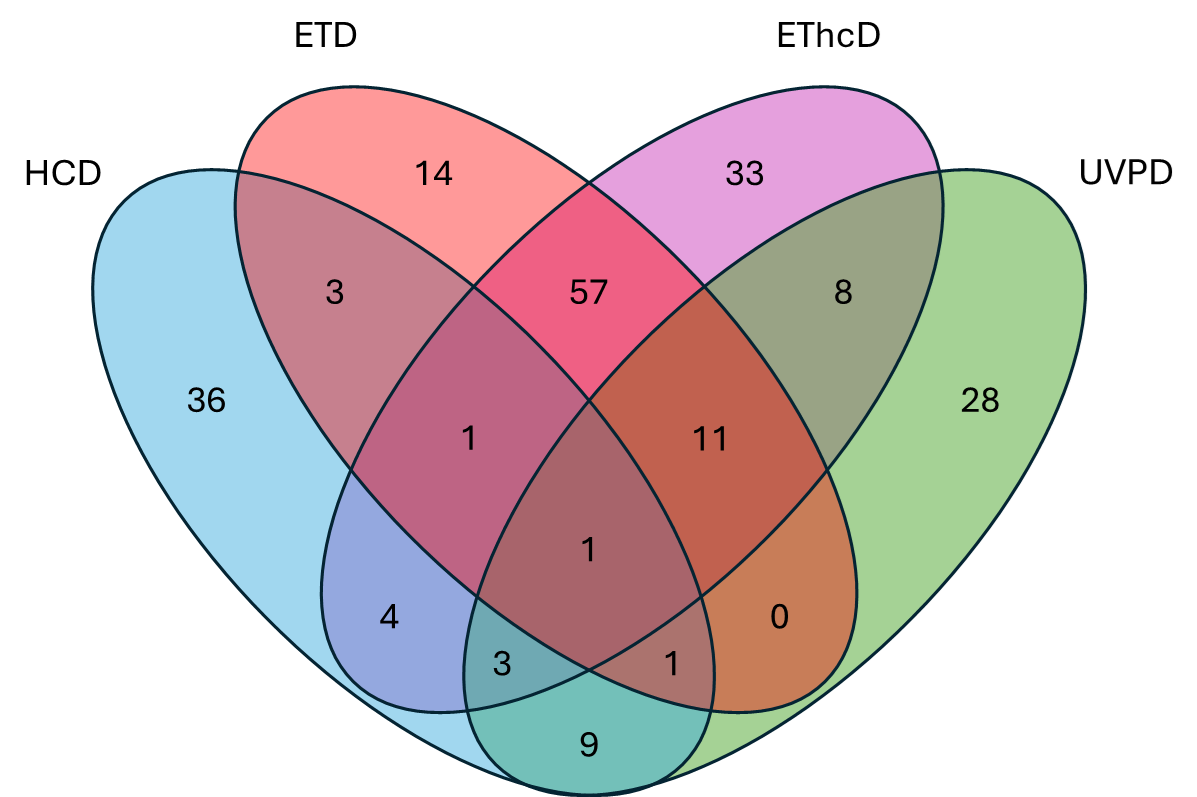


**Supplementary Figure 2:** Venn diagram representing the fragment ions identified for VP3 by each fragmentation techniques. A total of 209 fragment ions have been identified, representing a sequence coverage of 39%.

**
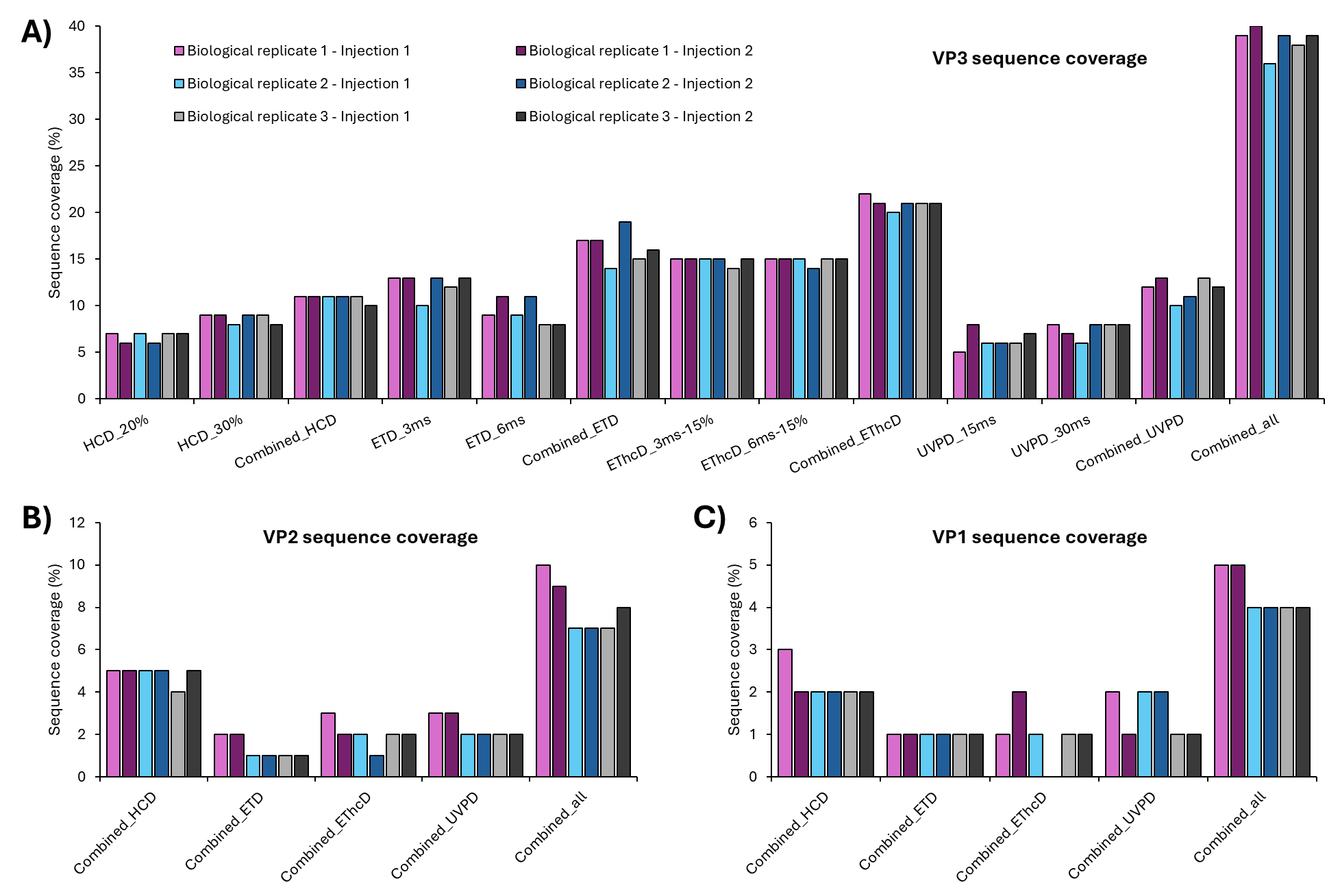
**

**Supplementary Figure 3:** Sequence coverage of (A) VP3, (B) VP2 and (C) VP1 for each replicate.


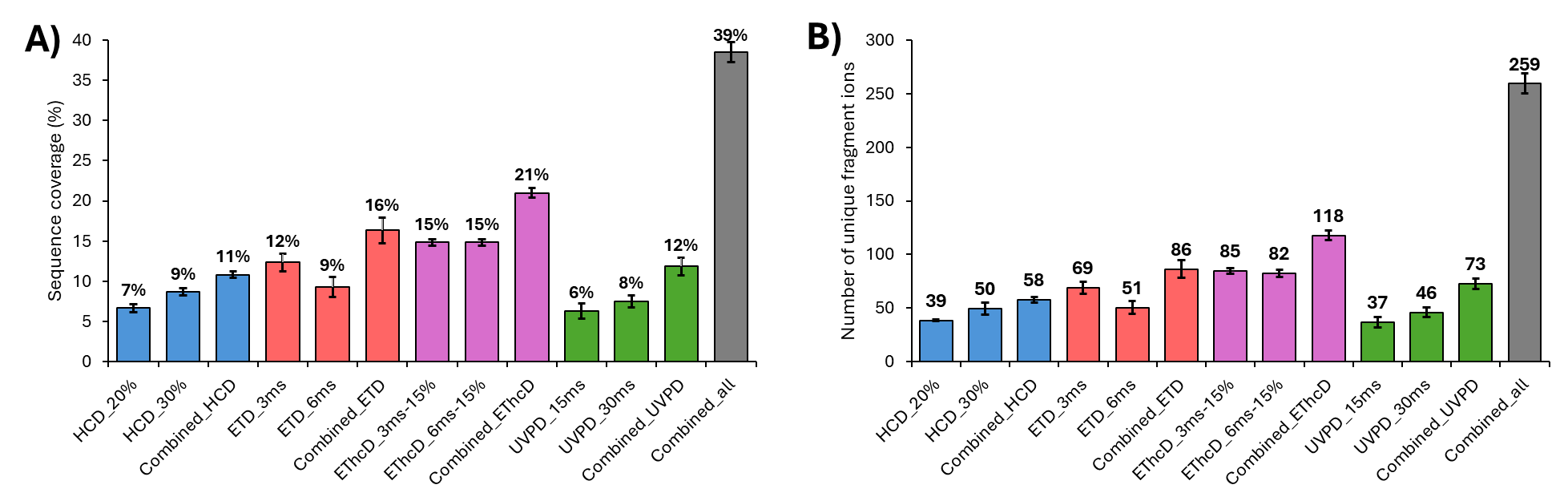


**Supplementary Figure 4:** (A) Average sequence coverage and (B) average number of unique fragment ions identified in each biological and injection replicates (n = 6, 3 biological replicates and 2 injection replicates for each) for all the fragmentation techniques and their combination.
